# Predictive Modeling of Kudzu (*Pueraria montana*) Habitat in the Great Lakes Basin of the United States

**DOI:** 10.3390/plants12010216

**Published:** 2023-01-03

**Authors:** Ashley M. Kovach-Hammons, Jordan M. Marshall

**Affiliations:** Department of Biological Sciences, Purdue University Fort Wayne, Fort Wayne, IN 46805, USA

**Keywords:** species distribution model, SDM, kudzu, invasive, maximum entropy, great lakes

## Abstract

Kudzu (*Pueraria montana* [Lour.] Merr. var. *lobata* [Willd.] Maesen & S.M. Almeida ex Sanjappa & Predeep) is an invasive woody vine widespread throughout much of the southeastern United States. New occurrences and recent studies using climatic parameters suggest that the Midwestern region of the United States is at the greatest risk of kudzu invasion. As there are already multiple reports of kudzu within the Great Lakes basin and no previous landscape models exist specifically for the basin, we developed probability models from existing spatial data (forest type, geology, land cover, precipitation, temperature, and known kudzu locations) by using maximum entropy methods at the national, regional, and basin scales. All three models had relatively high accuracy and strong positive correlation between predicted and observed values. Based on evaluation of the models using a testing data set, we determined a presence threshold and categorized areas within each model as suitable or unsuitable habitat. We pooled the models and calculated mean habitat suitability within the Great Lakes basin. Much of the southern half of the basin was suitable for kudzu. Continuing management and further monitoring of kudzu spread are likely necessary to limit further introduction and mitigate spread of kudzu within the Great Lakes region.

## 1. Introduction

Over the second half of the 20th century, the global rate of biological diversity loss has increased, correlated with human activity, land use changes, and invasive species [1]. Natural diversity levels are crucial for ecosystem processes and services, and can impact the well-being, health, and security of humans through these processes and services [1]. To reduce this rate of biological diversity loss and to preserve native flora and fauna, it is important to monitor threats to diversity, like invasive species and habitat fragmentation, and to invoke management plans to preserve native species. Within the United States, not only do invasive species pose threats to native diversity, they also can cost upwards of $120 billion USD per year to manage, with upwards of $33 billion USD allocated specifically towards invasive plant management [2]. These costs may increase year-by-year as additional invasive plant species are introduced, compounded with the spread of established populations. Furthermore, invasive species can impact existing communities by out-competing native species, ultimately displacing them [2]. Invasive species can also impact ecosystem productivity, ecosystem mitigation of natural disasters and habitat degradation, and even human health through novel pathogen introductions [3]. Understanding invasive species and monitoring their spread are crucial in reducing the rate of biological diversity loss and their environmental and economic impacts.

One of the most prevalent invasive plant species in the United States is kudzu (*Pueraria montana* [Lour.] Merr. var. *lobata* [Willd.] Maesen & S.M. Almeida ex Sanjappa & Predeep), a woody vine native to eastern Asia [4]. Initially introduced as an ornamental into North America in 1876 as part of the Japanese gardens in the World’s Fair, kudzu has since had multiple intentional introductions for ornamental use, fodder, and erosion control [5,6,7,8]. Since these introductions, kudzu has established and spread to over three million hectares within the United States, with reports in 32 states [9,10]. Kudzu is widely established throughout the southeastern United States, but populations of kudzu have also established along the east coast and as far northwest as Washington State [10].

Kudzu alone is estimated to cost upwards of $336 million USD each year in losses, primarily due to its toll on forest productivity, with additional costs of approximately $81 USD per hectare per year for management within the United States [11]. In addition to monetary implications, kudzu poses ecological costs as well. Kudzu is fast-growing, able to grow up to 30 cm per day and up to 30 m per stem [4,12]. Due to this fast-growing nature, kudzu can cause losses in forest productivity and pose threats to local species diversity by growing over and shading out other plants [13,14]. Additionally, kudzu can climb existing vegetation and bridge tree canopies together, which may exacerbate the effects of windthrow and fire [14,15]. Kudzu also has an extensive and hardy root system and primarily reproduces asexually by growing new crowns and roots at the nodes of existing vines [8,14,16,17]. These growth habits coupled with the lack of natural pests and pathogens within the United States make kudzu difficult to eradicate once it invades.

Kudzu’s optimal climate was previously defined as mild winters and hot summers [4], and kudzu was believed to have a cold threshold of −20 °C [18]. However, recent studies suggest that this threshold may vary due to cold acclimation and therefore it may survive further north than previously expected [18]. This is of particular concern as kudzu has already been reported in multiple locations in Michigan, some as far north as Benzie County (USDA Hardiness Zones 5b, 6a, and 6b) [19]. As kudzu may be able to withstand colder temperatures than initially thought, management may be necessary to control these populations. For example, Benzie County has experienced minimum temperatures at or below −20 °C in 56 of the last 120 years, as well as 13 of the last 30 years [20].

Though species distribution models (SDM) were first used in the 1920s, they have become increasingly popular within the past few decades to help understand changes in individual species ranges and biodiversity patterns, as well as to predict invasive species distributions [21]. There are many approaches to species distribution modeling, but climatic approaches are more common today with the heightened concern for how climate change can influence future distributions and for conservation of species of concern [21]. Maximum entropy models have become increasingly popular in species distribution modeling as they include machine-learning concepts and can account for more complicated parameters and interactions among those parameters [22,23]. In fact, Elith et al. [22] and Baldwin et al. [24] compared maximum entropy models to the well-established generalized linear model, generalized additive model, and bioclimate envelope model techniques and both studies found that maximum entropy outperformed these other model types. Maximum entropy was found to be particularly advantageous with limited presence locations and with more complicated parameters [22,24]. Species distribution models have previously been used to predict kudzu distributions across the United States.

Bradley et al. [25] used bioclimate envelope and maximum entropy modeling techniques with future climate data predictions to determine invasion risk for kudzu, privet (*Ligustrum sinense* Lour. and *L. vulgare* L.), and cogongrass (*Imperata cylindrica* [L.] P. Beauv.) in the eastern United States. Furthermore, Callen and Miller [9] used maximum entropy modeling techniques based on climate variables to compare kudzu’s native range with suitable habitat within the United States, while these studies focused on the United States at the national scale, few studies have sought to model kudzu distribution at the landscape scale, and there are no available studies that assess kudzu habitat suitability at the landscape scale for the Great Lakes basin, which is within the high-risk Midwestern United States. Kudzu has already been reported within at least twelve counties within the Great Lakes basin across Illinois, Indiana, Michigan, and Ohio [10], and new studies are finding that this region is at high risk for kudzu invasion [9,25].

The objectives of this study were (1) to predict kudzu habitat suitability within the United States portion of the Great Lakes basin based on national, regional, and basin scale maximum entropy models and (2) to compare the habitat suitability predicted across these scales.

## 2. Results

Based on pairs plot, we retained all independent variables for our maximum entropy model development (Figure S1). The national scale kudzu locations were reduced by 30.7% by removing duplicates in the ’maxent’ function (Table 1). The regional scale locations were reduced by 16.7%, whereas the basin scale locations were reduced by 9.1% (Table 1). Precipitation was the largest contributor to the national scale model, which had dramatically less influence in the regional and basin scale models (Table 1). Temperature was the most influential in the regional and basin models, with land cover type (NLCD) increasing in both models as spatial scale decreased (Table 1), while it was retained, geology was a minor contributor to all three models (Table 1).

**Figure 1:**
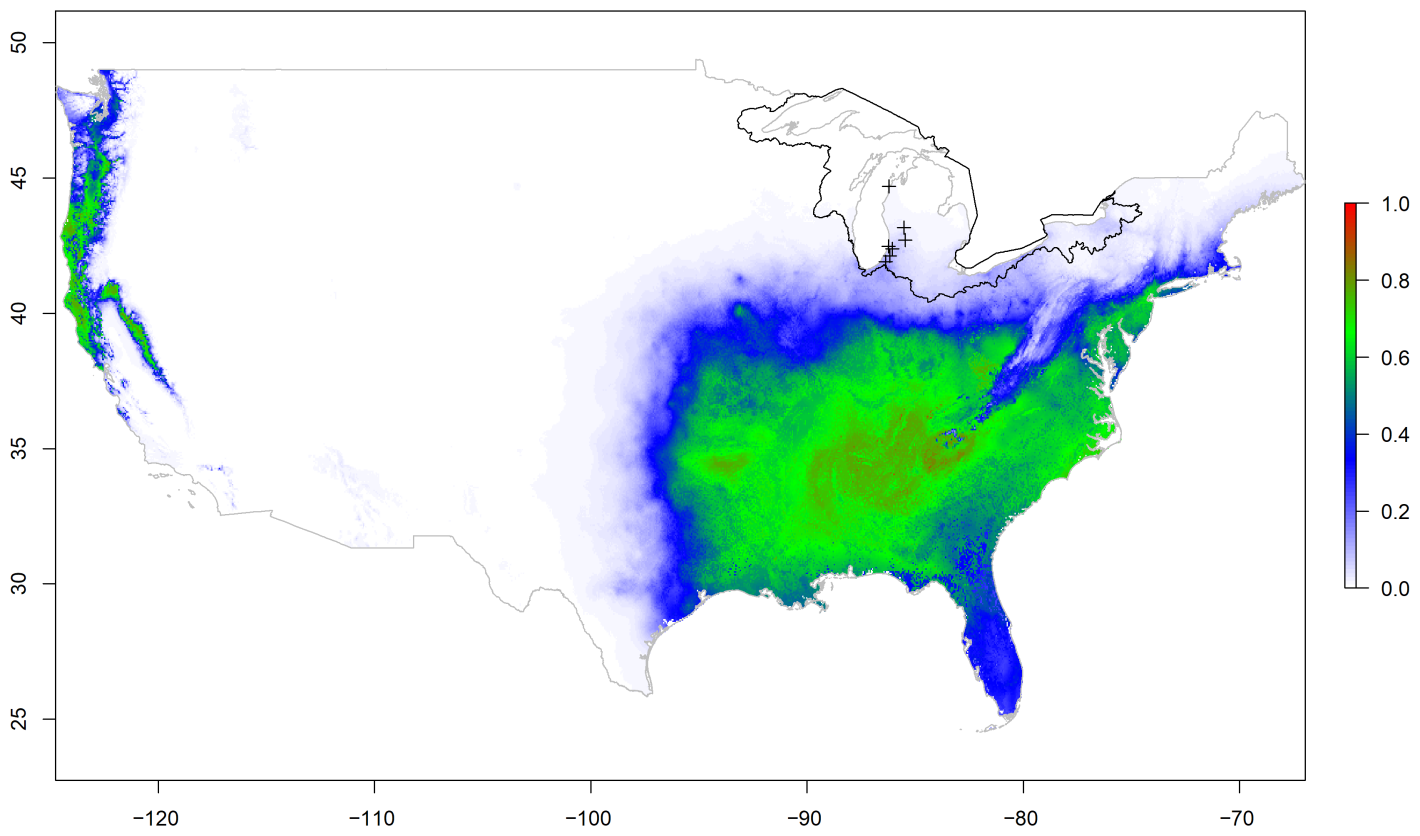
Maximum entropy model for kudzu probability based on forest type, geological parent material, National Land Cover Database categories, total annual precipitation, slope, and mean annual temperature in the conterminous United States. Black polygon indicates the Great Lakes basin boundary in the United States. Plus symbols represent kudzu locations used for model testing within the Great Lakes basin.

**Figure 2:**
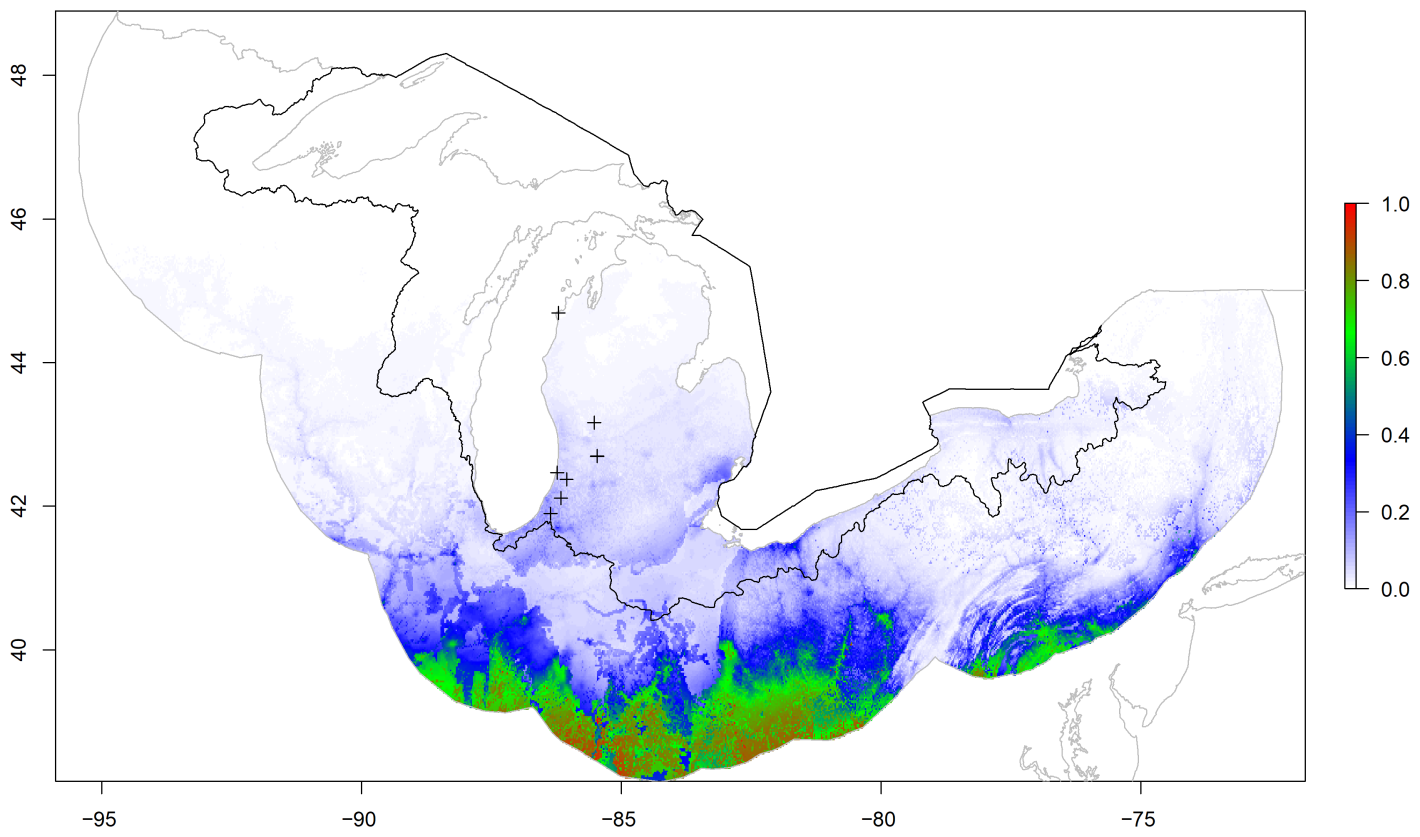
Maximum entropy model for kudzu probability based on forest type, geological parent material, National Land Cover Database categories, total annual precipitation, and mean annual temperature in a regional buffer (2.25 arc-seconds) around the Great Lakes basin boundary in the United States (black polygon). Plus symbols represent kudzu locations used for model testing within the Great Lakes basin.

**Figure 3:**
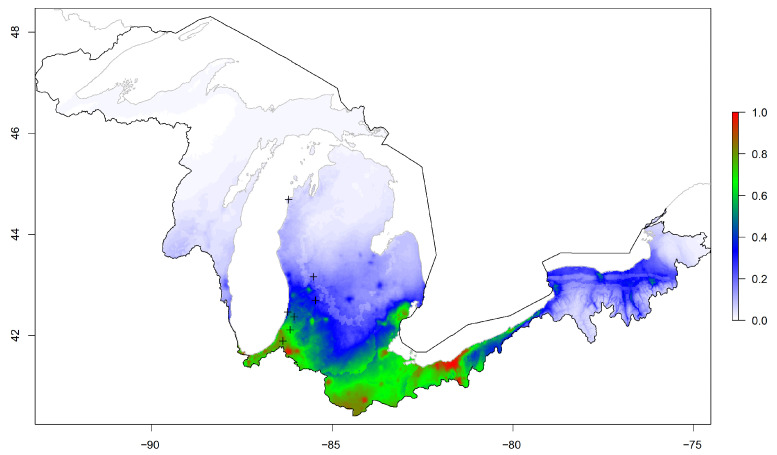
Maximum entropy model for kudzu probability based on forest type, geological parent material, National Land Cover Database categories, total annual precipitation, and mean annual temperature in the Great Lakes basin in the United States (black polygon). Plus symbols represent kudzu locations used for model testing within the Great Lakes basin.

**Figure 4:**
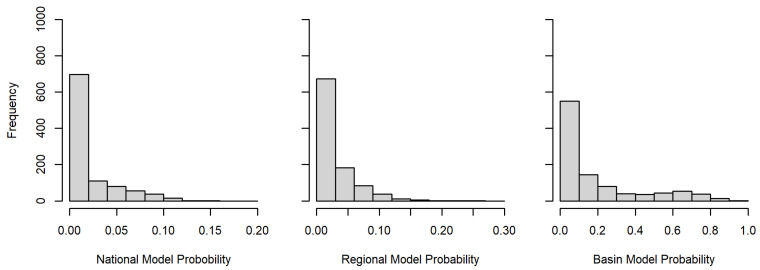
Histogram of national, regional, and basin scale model probabilities for 1000 random locations within the Great Lakes basin.

The national and regional models produced relatively low probabilities of kudzu within the Great Lakes basin (Figure 1 and Figure 2). Alternatively, the basin model provided areas with relatively high and low probabilities for kudzu within the Great Lakes basin boundary (Figure 3). This visual comparison was supported with significant differences between the three models for 1000 random locations within the basin (F${}_{2,2997}$ = 465.7, *p* < 0.001). The national and regional probabilities did not differ (national = 0.020 ± 0.027, regional = 0.026 ± 0.034; *p* = 0.573). However, the basin mean probability was significant greater than the other two models (basin = 0.185 ± 0.233; vs. national *p* < 0.001; vs. regional *p* < 0.001), while the frequency histograms for all three models were skewed right, neither the national nor the regional model probabilities exceeded 0.25 (Figure 4).

Each presence and absence location was defined as a true or false positive or negative location based on the κ-value, the threshold was set at 0.007 for the national model, where κ was the maximized kappa value from evaluating the model. The McNemar’s test for the national model rejected the null hypothesis that the false positive and false negative values were equal (χ^2^ = 4.17, *p* = 0.041), as there were zero false negative locations. Additionally, the accuracy for the model was 0.80, the Matthew’s Correlation Coefficient (MCC) was 0.65, and the area under the receiver operating characteristic curve (AUC) was 0.804. Of the 1000 random locations within the Great Lakes basin (Figure 4), 449 locations were ≥κ in the national scale model.

The regional scale model test resulted in a κ-value of 0.048 with the McNemar’s test failing to reject the null hypothesis that the false positive (=2) and false negative (=4) values were equal (χ^2^ = 0.17, *p* = 0.683). The AUC was slighly lower than the national scale.

Model (0.782), but accuracy and MCC values (0.80 and 0.61, respectively) were similar to the national scale model. Additionally, of the 1000 random locations within the basin (Figure 4), 181 locations were ≥κ in the regional scale model.

The basin scale model test resulted in a κ-value of 0.171 with the McNemar’s test failing to reject the null hypothesis that the false positve (=4) and false negative (=3) values were equal (χ^2^ = 1.00, *p* = 1.000). Additionally, the accuracy and MCC values (0.77 and 0.53, respectively) were less than the other two models, while the AUC was comparable to both (0.796). Of the 1000 random locations within the basin (Figure 4), 323 locations were ≥κ in the basin scale model.

**Figure 5:**
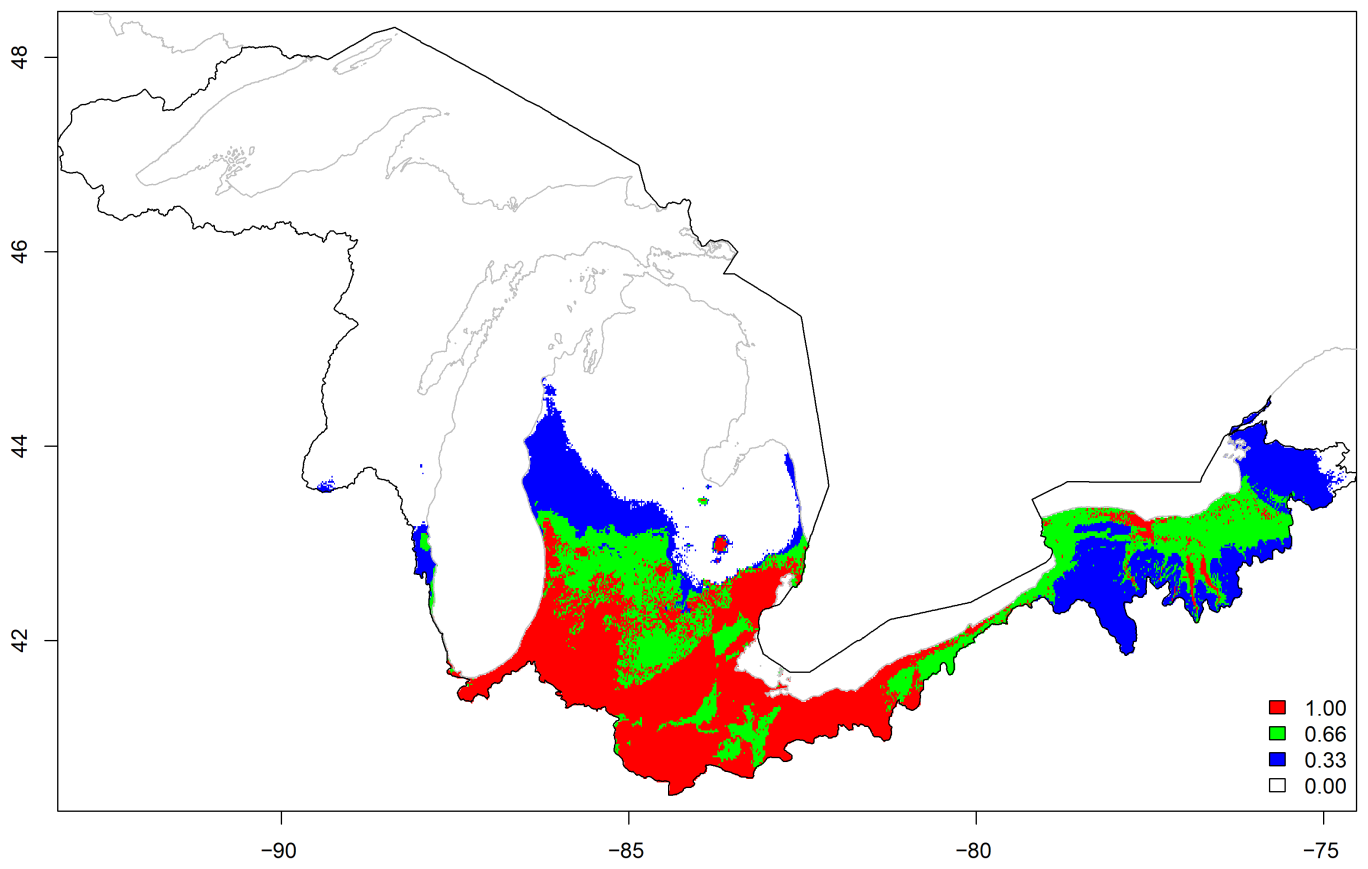
Mean likelihood of suitable habitat for kudzu in the Great Lakes basin (black polygon) in the United States from national, regional, and basin maximum entropy models. Suitable habitat was defined as ≥κ, where κ was the maximized kappa threshold for each model.

Finally, we reclassified pixels suitable (≥κ = 1) or unsuitable (<κ = 0) based on each model probabilities of kudzu presence and κ-values within the Great Lakes basin. The mean value map illustrated a high likelihood of kudzu habitat throughout much of the southern half of the Great Lakes basin (Figure 5). However, there are also areas of potentially suitable habitat extending north (Figure 5).

## 3. Discussion

Kudzu is a plant species introduced to North America and has spread throughout much of the eastern half of the United States causing various ecological and economic impacts since its importation in the late 1800s [4]. Past predictions of potential kudzu habitat in the United States resulted in relatively low risk categories within the Great Lakes basin [25]. These low probability or low risk predictions occur with 100-year climate change predictions (i.e., year 2100), where current climate conditions result in kudzu models with northern limits well south of the species current distribution [25]. Suitable habitat predictions within the Great Lakes basin based on the native range in Asia for calibration resulted in relatively small areas in the southern portions of the basin defined as suitable habitat [9]. Even incorporating forest types, geological features, and land cover, with current temperature and precipitation, we observed relatively low probability values within the Great Lakes basin. However, by utilizing the maximized κ as our presence and absence threshold, we did identify large areas of the southern and central portions of the Great Lakes basin as suitable for kudzu.

The models we present in this study demonstrate the difficulty of predicting suitable habitat for a species with a broad fundamental niche and wide geographic range. Vázquez [26] suggested that niche breadth positively influences invasion success in plants. Niche breadth can be estimated in relation to the size of native range or number of different native habitats the species occupies [26]. In this case, kudzu evidently occupies a broad niche. Much of the conterminous United States has climatic conditions similar to the native range of kudzu [9]. The introduced range for kudzu (currently) extends from 25.6 °N in Florida to 45.7 °N in Oregon. Within the Great Lakes basin, the northern most location is at 44.7 °N in Benzie County, Michigan. As much of the eastern half of the United States has climate conditions highly similar to the native range of kudzu [9], it likely has evolved a broad niche to cope with very different local climates spanning 20 degrees of latitude. This broad range of suitable climate in its native range (and subsequently across much of the eastern United States as an introduced range) resulted in relatively low probability values from our maximum entropy models in the Great Lakes basin. Our kudzu testing locations in those low probability areas then set our presence threshold (i.e, κ) lower than an arbitrary threshold that is often selected and typically unreliable (e.g., 0.5) [27]. As previous studies had focused on climate with temperature and precipitation [9,25], we tried to improve prediction of kudzu habitat in the Great Lakes basin by incorporating edaphic (i.e., geology), anthropogenic (i.e., NLCD), and ecological interaction (i.e., forest type) variables.

Previously, Coiner et al. [18] suggested that kudzu may acclimate to colder temperatures than previously believed, complicating the definition of a northern distribution limit for kudzu invasion based on geography and cold temperature thresholds alone, while our study employs 30-year averages for temperature and precipitation, extreme weather events may also impact kudzu survival and are likely to become more intense and frequent with climate change [28]. The impacts of these extreme weather events will also vary based on when they occur. For example, extreme cold temperatures (e.g., frosts) that occur towards the end of the growing season would be more detrimental to kudzu than the same extreme cold temperatures during winter months, as kudzu would not yet be acclimated to such temperatures during the growing season [18,29]. Moisture balance and photoperiod regimen may also explain differences in woody plant acclimation to colder temperatures across larger scales [29,30]. This study focuses on predicting habitat suitability for kudzu at different scales using long-term climatic variables, land cover and forest types, and geology. Future studies on kudzu habitat distributions may want to additionally consider how these extreme weather events and moisture balance impact local kudzu populations’ mortality and spread.

While the actual probabilities of occurrence from our maximum entropy models were similar to other kudzu modeling studies (e.g., [9,25]), we observed suitable habitat for kudzu in a relatively large proportion of the Great Lakes basin. As there are few currently known infestation locations (i.e., plus symbols in Figure 3), unoccupied, available habitat within the basin should be of concern to natural resource managers. Kudzu primarily reproduces through clonal reproduction, which requires existing crowns of kudzu plants [4,15,17,31]. The disjunct nature of kudzu records currently in the Great Lakes basin exemplifies the importance of human transport for kudzu invasion. We argue that this makes invasion by kudzu into the Great Lakes basin dispersal-limited as much of the suitable habitat is not occupied; however, the introduction of individuals will lead to establishment [32]. Likely, kudzu is not habitat-limited in the Great Lakes basin, specifically in the southern half, due to the species niche breadth.

## 4. Materials and Methods

Maximum entropy models were developed to predict the probability of kudzu habitat suitability in the United States based on climatic and physical environmental variables. Independent variables for model development included total annual precipitation (mm), mean annual temperature (°C), land cover and forest types, and geology (parent material). Prior to model development, a pairs plot was used to identify collinear independent variables for potential omission. Total annual precipitation was derived as the sum of 12 months of 30-year (1970–2000) historical climate data at a spatial resolution of 30 arc-seconds (~1 km^2^) [33], which became the base resolution for rasterizing vector data and resampling existing rasters via median calculations. Mean annual temperature was derived as the mean of 12 months of 30-year historical climate data at a spatial resolution of 30 arc-seconds [33]. Land cover was the U.S. Geological Survey National Land Cover Database for 2019 [34]. Forest types were derived from Forest Inventory and Analysis data [35]. Geology was rasterized from the geodatabase of the conterminous United States polygons representing geologic features [36]. Global climate data and United States data were clipped to the conterminous US boundary.

We used the ‘maxent’ function within the package *dismo* (version 1.3-9) [37] with jackknifing in R as a wrapper for Maxent Software (version 3.4.4) [22]. Forest types, geology, and land cover types were included as categorical factors. Precipitation and temperature were included as continuous data. Known kudzu locations were used as presence locations for model development [10]. The national scale data set included 6152 kudzu locations within the conterminous United States. We used the default of 10,000 random background locations for the national scale model. The regional scale data set included 197 kudzu locations and spatially included a 2.25 arc-seconds buffer around the Great Lakes basin. As the number of background locations will influence model predictions [38], we scaled the number of random background locations proportionally by the spatial scale for both the regional and basin scale models (Table 1). The basin scale data set included 11 kudzu locations and spatially included the hydrologically defined Great Lakes basin. We included the option ‘removeDuplicates = T’ in the ‘maxent’ function to reduce bias where location points occurred with the same independent variable pixel values. We selected 1000 random locations within the Great Lakes basin boundary and extracted the three model probabilities. We used analysis of variance (ANOVA) with Tukey’s HSD as a post hoc test to compare the probabilities across the basin between models [39].

To test the models and calculate true positive (observed and predicted presence), known kudzu locations from the Midwest Invasive Species Information Network were used within the Great Lakes basin [40], while this data set is relatively small (n = 15), it provided a suitable testing set of what is known within the Great Lakes basin. Further, to calculate true absence (observed and predicted absence), we randomly selected an additional 15 locations within the Great Lakes basin to serve as “absence” locations. We evaluated the maximum entropy models, using the ’evaluate’ function in *dismo* package in R to calculate area under the receiver operating characteristic curve (AUC) and the maximized kappa (κ) as our predictive presence threshold [26]. At the 30 locations (presence and absence), we extracted the probability for each model and applied the predicted presence threshold of κ (i.e., if the predicted probability was ≥κ, it was coded as a predicted presence). This allowed us to calculate true positive (observed and predicted presence) and true negative (observed and predicted absence) counts, as well as false positive (observed absence but predicted presence) and false negative (observed presence but predicted absence) counts. From these count values, we used a McNemar test for the hypothesis that the false negative and false positive values were equal [41]. Additionally, we calculated accuracy as:

(1) $$Accuracy=\frac{TP+TN}{TP+TN+FP+FN}$$

where TP was true positive, TN wass true negative, FP was false positive, and FN was false negative. Accuracy aided in characterizing the overall model success. Finally, we calculated a Matthew’s correlation coefficient (MCC) [42] to test the correlation between observed and predicted values as:

(2) $$MCC=\frac{TP*TN-FP*FN}{\sqrt{\left(TP+FP)*(TP+FN)*(TN+FP)*(TN+FN\right)}}$$

Finally, we coded habitat as suitable and unsuitable within the Great Lakes basin for each model (cropped to the basin boundary). If a pixel was ≥κ, then it was coded as suitable (1), and if it was <κ, then it was coded as unsuitable (0). We calculated mean suitability within the Great Lakes basin as a mean pixel value of the pooled models.

## Figures and Tables

**Table 1:** National, regional, and Great Lakes basin scale maximum entropy model independent variable percent contribution, geographic area modeled, number of kudzu presence locations used for model training (after duplicate values were removed), number of random background locations used, and area under the receiver operating characteristic curve (AUC). Forest type, Geology (parent material ID code) and NLCD (National Land Cover Database category code) were included as categorical factors. Precipitation (mm) was annual total and Temperature (°C) was annual mean, both from 30 to year historical data.

Variable	National	Regional	Basin
Forest	1.3	4.6	0.0
Geology	0.2	3.6	1.0
NLCD	0.0	2.2	7.4
Precipitation	81.3	0.4	1.2
Temperature	17.2	89.3	90.5
Area (km^2^)	7.80 × 10^6^	1.12 × 10^6^	4.48 × 10^5^
Kudzu Locations	4263	164	10
Background	10,000	1436	574
AUC	0.795	0.906	0.900

## Data Availability

Data available from original sources.

## Supplementary Materials

The following supporting information can be downloaded at: https://www.mdpi.com/article/10.3390/plants12010216/s1, Figure S1: Pairs plot between forest type, geological parent material, National Land Cover Database, total annual precipitation, and mean annual temperature.

## References

[1] Millennium Ecosystem Assessment (2005). Ecosystems and Human Well-Being: Biodiversity Synthesis.

[2] Pimentel D., Zungia R., Morrison D. (2005). Update on the environmental and economic costs associated with alien-invasive species in the United States. Ecol. Model.

[3] Lodge D.M., Williams S., MacIssac H.J., Hayes K.R., Leung B., Reichard S., Mack R.N., Moyle P.B., Smith M., Andow D.A. (2006). Biological invasions: Recommendations for US policy and management. Ecol. Appl..

[4] Lindgren C.J., Castro K.L., Coiner H.A., Nurse R.E., Darbyshire S.J. (2013). The biology of invasive alien plants in Canada. 12. *Pueraria montana* var. *lobata* (Willd.) Sanjappa & Predeep. Can. J. Plant Sci..

[5] Brown K.H. (2008). Fair Japan: Japanese gardens at American World’s Fairs, 1876–1940. SiteLINES.

[6] Guertin P.J., Denight M.L., Gebhart D.L., Nelson L. Invasive species biology, control, and research. Part 1: Kudzu (*Pueraria montana*). Engineer Research and Development Center. US Army Corps of Engineers. https://apps.dtic.mil/sti/pdfs/ADA491410.pdf.

[7] Pieters A.J. (1932). Kudzu, a Forage Crop for the Southeast.

[8] Winberry J.J., Jones D.M. (1973). Rise and decline of the “miracle vine”: Kudzu in the southern landscape. Southeast Geogr..

[9] Callen S.T., Miller A.J. (2015). Signatures of niche conservatism and niche shift in the North American kudzu (*Pueraria montana*) invasion. Divers. Distrib..

[10] EDDMapS Early Detection & Distribution Mapping System. The University of Georgia—Center for Invasive Species and Ecosystem Health. http://www.eddmaps.org/.

[11] Boyette C.D., Walker H.L., Abbas H.K. (2002). Biological control of kudzu (*Pueraria lobata*) with an isolate of *Myrothecium verrucaria*. Biocontrol Sci. Technol..

[12] Mitich L.W. (2000). Intriguing World of Weeds. Kudzu [*Pueraria lobata* (Willd.) Ohwi]. Weed Technol..

[13] Britton K.O., Orr D., Sun J., Van Driesche R., Lyon S., Blossey B., Hoddle M., Reardon R. (2002). Kudzu. Biological Control of Invasive Plants in Eastern United States.

[14] Forseth I.N., Innis A.F. (2004). Kudzu (*Pueraria montana*): History, physiology, and ecology combine to make a major ecosystem threat. Crit. Rev. Plant Sci..

[15] Munger G.T. *Pueraria montana* var. *lobata*. Fire Effects Information System. USDA Forest Service, Rocky Mountain Research Station, Fire Sciences Laboratory. https://www.fs.usda.gov/database/feis/plants/vine/puemonl/all.html.

[16] Abramovitz J.N. (1983). *Pueraria lobata* Willd. (OHWI), Kudzu: Limitations to Sexual Reproduction. Master’s Thesis.

[17] Bentley K.E., Mauricio R. (2016). High degree of clonal reproduction and lack of large-scale geographic patterning mark the introduced range of the invasive vine, kudzu (*Pueraria montana* var. *lobata*), in North America. Am. J. Bot..

[18] Coiner H.A., Hayhoe K., Ziska L.H., Van Dorn J., Sage R.F. (2018). Tolerance of subzero winter cold in kudzu (*Pueraria montana* var. *lobata*). Oecologia.

[19] USDA Plant Hardiness Zone Map. Agricultural Research Service, U.S. Department of Agriculture. https://planthardiness.ars.usda.gov/.

[20] NOAA Climate Data Online: Benzie County, MI (FIPS:26019). National Centers for Environmental Information. https://www.ncdc.noaa.gov/cdo-web/datasets/GHCND/locations/FIPS:26019/detail.

[21] Guisan A., Thuiller W. (2005). Predicting species distribution: Offering more than simple habitat models. Ecol. Lett..

[22] Elith J., Graham C.H., Anderson R.P., Dudík M., Ferrier S., Guisan A., Hijmans R.J., Huettmann F., Leathwick J.R., Lehmann A. (2006). Novel methods improve prediction of species’ distributions from occurrence data. Ecography.

[23] Gastón A., García-Viñas J.I. (2011). Modelling species distributions with penalized logistic regressions: A comparison with maximum entropy models. Ecol. Model.

[24] Baldwin R.A. (2009). Use of maximum entropy modeling in wildlife research. Entropy.

[25] Bradley B.A., Wilcove D.S., Oppenheimer M. (2010). Climate change increases risk of plant invasion in the Eastern United States. Biol. Invasions.

[26] Vázquez D.P., Cadotte M.W., McMahon S.M., Fukami T. (2006). Exploring the relationship between niche breadth and invasion success. Conceptual Ecology and Invasion Biology.

[27] Freeman E.A., Moisen G.G. (2008). A comparison of the performance of threshold criteria for binary classification in terms of predicted prevalence and kappa. Ecol. Model.

[28] Pörtner H.-O., Roberts D.C., Tignor M., Poloczanska E.S., Mintenbeck K., Alegría A., Craig M., Langsdorf S., Löschke S. (2022). Climate Change 2022: Impacts, Adaptation and Vulnerability. Contribution of Working Group II to the Sixth Assessment Report of the Intergovernmental Panel on Climate Change.

[29] Widrlechner M.P., Daly C., Keller M., Kaplan K. (2012). Horticultural applications of a newly revised USDA Plant Hardiness Zone Map. HortTechnology.

[30] Widrlechner M.P. (1994). Environmental analogs in the stress-tolerant landscape plants. J. Arboric..

[31] Geerts S., Mashele B.V., Visser V., Wilson J.R.U. (2016). Lack of human-assisted dispersal means *Pueraria montana* var. *lobata* (kudzu vine) could still be eradicated from South Africa. Biol. Invasions.

[32] Ehrlén J., Eriksson O. (2000). Dispersal limitation and patch occupancy in forest herbs. Ecology.

[33] Fick S.E., Hijmans R.J. (2017). WorldClim 2: New 1-km spatial resolution climate surfaces for global land areas. Int. J. Climatol..

[34] Dewitz J. National Land Cover Database (NLCD) 2019 Products. US Geological Survey.

[35] Ruefenacht B., Finco M.V., Nelson M.D., Czaplewski R., Helmer E.H., Blackard J.A., Holden G.R., Lister A.J., Salajanu D., Weyermann D. (2008). Conterminous U.S. and Alaska forest type mapping using Forest Inventory Analysis data. Photogramm. Eng. Remote Sens..

[36] Horton J.D. The State Geologic Map Compilation (SGMC) Geodatabase of the Conterminous United States (ver. 1.1, August 2017), U.S. Geological Survey. https://www.sciencebase.gov/catalog/item/5888bf4fe4b05ccb964bab9d.

[37] Hijmans R.J., Phillips S., Leathwick J., Elith J. Dismo: Species Distribution Modeling. R Package Version 1.3-9. https://cran.r-project.org/packages=dismo.

[38] Merow C., Smith M.J., Silander S.A. (2013). A practical guide to MaxEnt for modeling species distributions: What it does, and why inputs and settings matter. Ecography.

[39] Sokal R.R., Rohlf F.J. (2012). Biometry: The Principles and Practice of Statistics in Biological Research.

[40] Midwest Invasive Species Information Network. Michigan State University. https://www.misin.msu.edu/.

[41] Pembury Smith M.Q.R., Ruxton G.D. (2020). Effective use of the McNemar test. Behav. Ecol. Sociobiol..

[42] Chicco D., Jurman G. (2020). The advantages of the Matthews correlation coefficient (MCC) over F1 score and accuracy in binary classification evaluation. BMC Genom..

