# Supplementary figures and images for: Predictive Modeling of Kudzu (Pueraria montana) Habitat in the Great Lakes Basin of the United States
Source: Plants (Basel). 2023 Jan 3;12(1):216. doi: 10.3390/plants12010216 (PMC9824185; doi:10.3390/plants12010216)

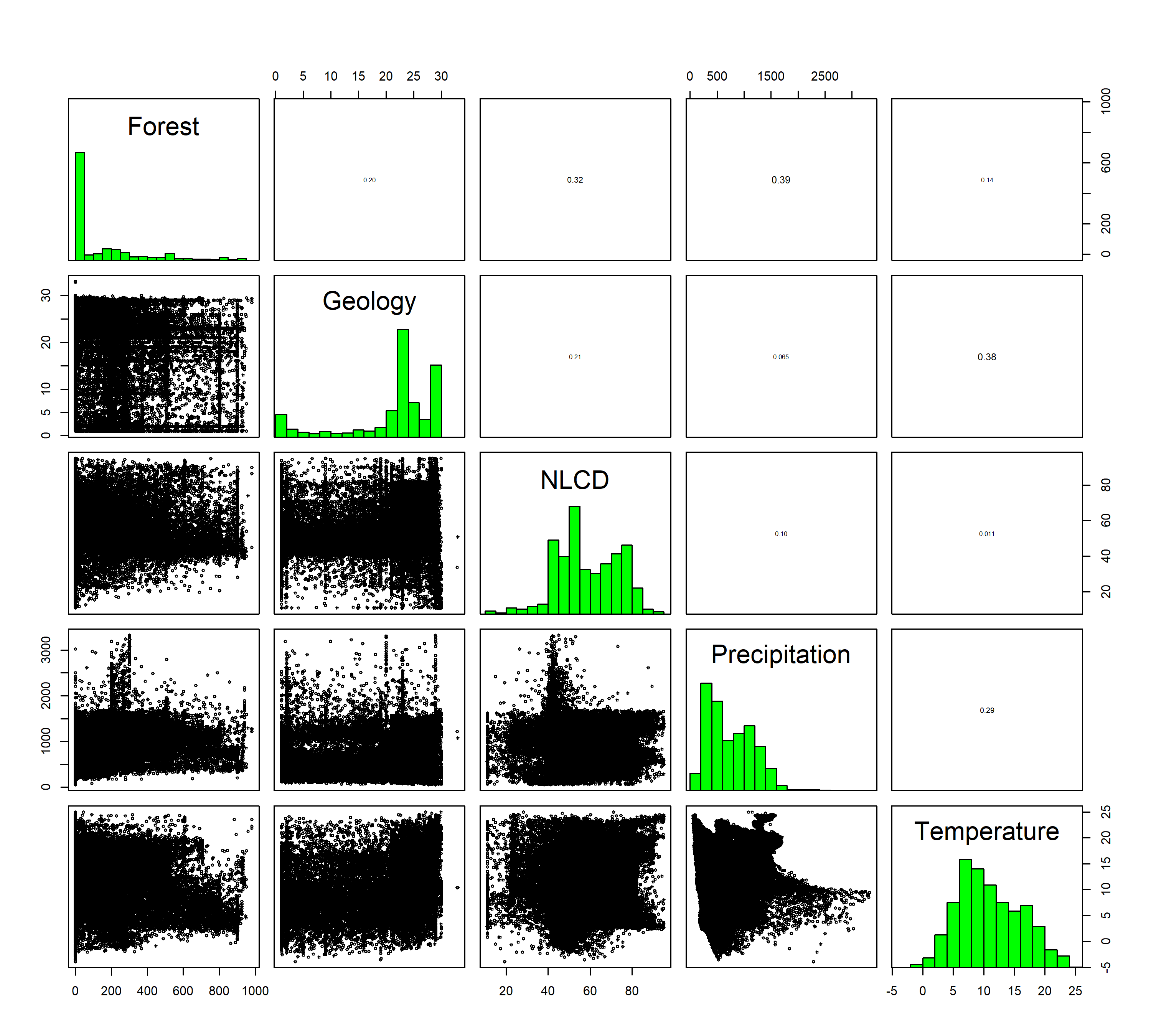

Supplement: Supplementary file 1 [file plants-12-00216-s001.zip › plants-2076621-supplementary.png]
